# A Comparative Analysis of *Bombyx mori* (Lepidoptera: Bombycidae) *β*-fructofuranosidase Homologs Reveals Different Post-Translational Regulations in *Glyphodes pyloalis* Walker (Lepidoptera: Pyralidae)

**DOI:** 10.3390/insects13050410

**Published:** 2022-04-26

**Authors:** Yue Zhao, Liangli Yang, Yu Chen, Xinwei Zhang, Jing Li, Dan Liang, Song Jiang, Junshan Gao, Yan Meng

**Affiliations:** 1 School of Life Sciences, Anhui Agricultural University, 130 West Changjiang Road, Hefei 230036, China; zhaoyue180421@163.com (Y.Z.); yliangli2019@163.com (L.Y.); 17356403916@163.com (Y.C.); 18725515051@163.com (X.Z.); lljj0617@163.com (J.L.); liangdanmolecule@163.com (D.L.); jiangsong2111@126.com (S.J.); gaojsh@ahau.edu.cn (J.G.); 2 Anhui International Joint Research and Development Center of Sericulture Resources Utilization, 130 West Changjiang Road, Hefei 230036, China; 3 Department of Pathology, Henan Provincial People’s Hospital, 7 Weiwu Road, Zhengzhou 450003, China

**Keywords:** *β*-fructofuranosidase, *Glyphodes pyloalis* Walker, mulberry feeding, N-glycosylation

## Abstract

**Simple Summary:**

The *β*-fructofuranosidase (*β*-FFase) encoding gene *BmSuc1* regulates the glycometabolism of silkworm larvae, and it participates in the resistance of mulberry alkaloids. However, there is no molecular or biochemical information available about the mulberry pest *Glyphodes* *pyloalis* Walker *β*-FFase homologs. In this paper, we have obtained five *β*-FFase homologous genes in *G. pyloalis* and characterized the expression and the localization of GpSUC1a in the midgut. The *β*-FFase activity in the midgut of *G. pyloalis* larvae and GpSUC1a were both confirmed, while recombinant GpSUC1a displayed little activity as compared with the higher activity of BmSUC1. Some putative N-glycosylation sites were found in GpSUC1a but none in BmSUC1, while there was more methylation in BmSUC1 than in GpSUC1a. The results indicate that such post-translational modifications (PTMs) are differentially supporting that *β*-FFase are active in these two mulberry feeding caterpillars, and the activation of GpSUC1a may be controlled by a more complex post-translational regulatory system in *G. pyloalis* larvae. This is the first report on the characterization of *β*-FFase genes from *G. pyloalis* and the first comparison of expression regulation between two mulberry feeding insects *B. mori* and *G. pyloalis*. Moreover, this research may provide new ideas for the management of mulberry borers.

**Abstract:**

The silk-spinning and Lepidopteran model insect *Bombyx mori* (Bombycidae) is a mulberry specialist. The *BmSuc1* gene is the first *β*-fructofuranosidase (*β*-FFase) encoding gene identified in animals, and *β*-FFase acts as an essential sucrase for glycometabolism modulation in the silkworm larvae, involved in resistance to mulberry alkaloids. *Glyphodes pyloalis* Walker (Lepidoptera: Pyralidae) is an important mulberry pest leading to heavy economic loss of sericulture. However, no molecular or biochemical information is available about *G. pyloalis β*-FFase homologs. In this study, five *β*-FFase homologous genes in *G. pyloalis* were obtained. The genes *GpSuc1a* and *GpSuc2c* were expressed in the midgut; *GpSuc2c* encodes a truncated polypeptide. The expression and the localization of GpSUC1a in the midgut was characterized. Whereas recombinant GpSUC1a expressed in both *Escherichia coli* and BmN cells displayed little activity as compared with higher activity of BmSUC1, *β*-FFase activity in the larval midgut of *G. pyloalis* and GpSUC1a purified from the midgut were both confirmed. The data suggested that the activation of GpSUC1a is probably controlled by a more complicated post-translational regulation system in *G. pyloalis* larvae than that of BmSUC1 in *B. mori*. To study post-translational modifications (PTMs), GpSUC1a and BmSUC1 were purified from larval midguts using immunoprecipitation and subjected to LC-MS to perform PTMs analysis. Some putative N-glycosylated sites were found in GpSUC1a but none in BmSUC1, while there was more methylation in BmSUC1 than in GpSUC1a, indicating that such PTMs were supporting the differential *β*-FFases activities in these two mulberry feeding caterpillars.

## 1. Introduction

Many plant species produce toxic compounds that serve as a defense mechanism against insect herbivores and pathogens [1,2,3,4]. Plant latex often contains a variety of toxic compounds, such as alkaloids and proteases, which provide a defense against insect herbivory [5,6,7,8]. Mulberry latex contains high concentrations of alkaloidal sugar mimics such as 1,4-dideoxy-1,4-imino-D-arabinitol (D-AB1) and 1-deoxynojirimycin (DNJ) [9,10]. Sugar-mimic alkaloids adversely affect the growth of non-mulberry phytophagous insects with low dose, such as the Eri silkworm, by interfering with sugar metabolism [11,12,13]. However, the silkworm *Bombyx mori* (Lepidoptera: Bombycidae), which is adapted to feeding on mulberry leaves, is unaffected by these toxins. *B. mori* has evolved mechanisms to avoid the toxicity of sugar-mimic alkaloids. These mechanisms enable it to use mulberry leaves for growth, probably by using a *β*-fructofuranosidase (*β*-FFase)-triggering enzymatic adaptation system [12,14,15].

Sucrose hydrolases can be divided into *α*-glucosidase (EC 3.2.1.20) and *β*-FFase (EC: 3.2.1.26) according to the enzyme hydrolyzing an *α*-glucosyl or *β*-fructosyl residue of the substrate. The former is found in essentially all the organisms, while *β*-FFase is rarely found in animals [16,17,18]. Although they do not exhibit inhibitory activity against *β*-FFase, D-AB1 and DNJ are strong inhibitors of *α*-glucosidase [9,14]. The first *β*-FFase gene discovered and cloned in macroscopic animals, *BmSuc1* is specifically expressed in the *B. mori* larval midgut and in the silk gland. The activity of BmSUC1 is not affected by DNJ, and it may play an important role in the silkworm mulberry enzyme adaptation system [14]. Our further study proved that BmSUC1 acts as an essential sucrase for glycometabolism modulation in the silkworm larvae [19].

*Glyphodes pyloalis* Walker (Lepidoptera: Pyralidae) is a widely distributed mulberry pest. This caterpillar not only causes serious reductions in mulberry leaf yields [20,21] but also damages sericulture by transmitting viruses to the silkworm [22]. Currently, using conventional insecticides are the principal means to control *G. pyloalis* populations. However, the excessive use of insecticides in mulberry growing regions has caused silkworm poisoning and environmental pollution, as well as increased *G. pyloalis* resistance to a variety of chemical insecticides [23]. As *G. pyloalis* has the same mulberry-feeding habit as *B. mori*, identification of *BmSuc1* homologous genes and functional characterization in *G. pyloalis* may provide a better understanding of the role of insect *β*-FFases and their regulation mechanism. This research analyzed the expression and the activity of *β*-FFases in the midgut of *G. pyloalis* larvae. We also expressed the recombinant proteins in *E. coli* and BmN cells and investigated their enzymatic properties in vitro. In addition, we made a polyclonal antibody to GpSUC1a, and we used it to study GpSUC1a expression by western blotting and immunofluorescence experiments. Immunoprecipitation and LC-MS analyses revealed the difference in the expression of GpSUC1a and BmSUC1 in vivo. To our knowledge, this is the first report on the characterization of *β*-FFase genes from *G. pyloalis* and the first comparison of expression regulation between two mulberry feeding insects, *B. mori* and *G. pyloalis*. Moreover, this research may provide new ideas for the management of mulberry borers.

## 2. Materials and Methods

### 2.1. Insects, Vectors, and Cell Lines

*B. mori* strain p50 was used in this study. *G. pyloalis* was collected in the mulberry field of Anhui Agricultural University. Larvae were reared on fresh mulberry leaves at 25–26 °C under a 12:12 h (L:D) photoperiod with 70% relative humidity. The silkworm ovary-derived BmN cells were routinely maintained at 27 °C in TC100 medium (Ximeijie, China) supplemented with 10% fetal bovine serum (ExCell, Shanghai, China) and 1% penicillin-streptomycin (Solarbio, Beijing, China) in our laboratory. The expression vector pET-24b was purchased from Invitrogen. The pET24b-*BmSuc1* recombinant plasmid and the pFastBac-dual vector are always reserved in our laboratory. The *E. coli* strain BL21 (DE3) cells and DH10Bac (BmNPV) cells were purchased from Transgene Biotech (Beijing, China).

### 2.2. Enzymatic Determination of β-FFase in Larval Tissues

To determine *β*-FFase activity, the final instar larvae of *G. pyloalis* and *B. mori* (day 3 of fifth instar) were dissected in phosphate buffer saline (PBS, pH 7.4) on ice. The PBS was purchased from Sangon Biotech (item number: B040100-0005) (Beijing, China). Total proteins were extracted from the larval midgut and the silk gland by using a One Step Animal Tissue Active Proteins Extraction Kit (Sangon Biotech, Shanghai, China). Protein concentrations were measured using the BCA Proteins Assay Kit (Sangon Biotech, Shanghai, China). The *β*-FFase activity was detected using the 3,5-dinitrosalicylic acid (DNS) method as described by Gan et al. (2018) [19]. The methods of dealing with tissues referred to Gan et al. (2018) [19]. The standard 200 µL reaction mixture contained 80 μg of total tissue proteins, 100 mM sucrose or raffinose, and 10 mM Britton–Robinson buffer (pH 7.0). In the case of using sucrose as a substrate, 100 mM DNJ was added in the reaction mixture to inhibit *α*-glucosidase activity. The sucrose substrate was incubated with Britton-Robinson buffer for 10 min at 30 °C, following the method of measuring enzyme activity described by Gan et al. (2018) [19].

### 2.3. Degenerate Polymerase Chain Reaction (Degenerate PCR)

The genomic DNA of *G. pyloalis* was extracted from larval midguts by the phenolchloroform method. To isolate identical genes in the genome, two sets of degenerate primers (Table 1) were designed based on the consensus regions among the deduced amino acid sequences of BmSUC1 and BmSUC2 [14], and several *β*-FFases of other organisms obtained from GenBank (http://www.ncbi.nlm.nih.gov, accessed on 1 October 2018). To amplify a portion of the gene fragments, genomic-degenerate PCR was performed using a thermal cycling program of 94 °C for 10 min and 40 cycles of 94 °C for 1 min, 50 °C for 30 s and 72 °C for 1 min, followed by an additional extension at 72 °C for 7 min.

### 2.4. Reverse Transcription PCR (RT-PCR) and Rapid Amplification of cDNA Ends (RACE)

Total RNA was extracted from the whole body or from various tissues of *G. pyloalis*, using the Animal Total RNA Isolation Kit (Sangon Biotech, Shanghai, China). To profile the transcriptional patterns of *GpSuc1a*, *GpSuc1b*, *GpSuc2a*, *GpSuc2b*, and *GpSuc2c*, first-strand cDNA was synthesized from 1 μg of total RNA extracted from different tissues in a 20 µL reaction mixture using the M-MuLV First Strand cDNA Synthesis Kit (Sangon Biotech, Shanghai, China). Semiquantitative RT-PCR was carried out using the 18S as the control to normalize mRNA expression levels. The PCR reactions were performed as follows: denaturation at 94 °C for 5 min, 28 cycles of 94 °C for 1 min, 53 °C for 50 s, and 72 °C for 1 min. Primers are listed in Table 1. PCR products were separated on 1% agarose gels. To amplify the cDNA ends of five genes, 3 µg of total RNA extracted from the whole body was subjected to RACE with the SMARTerTM RACE cDNA Amplification Kit (Clontech, Shanghai, China) as recommended by the manufacturer. The gene-specific primer and the nest gene-specific primer used for the 5′- and 3′-RACE were designed based on their genomic DNA sequences shown in Table 1.

### 2.5. Expression of Recombinant Proteins in E. coli and Preparation of GpSUC1a Polyclonal Antibody

Primers for amplifying the open reading frame (ORF) of *GpSuc1a*, *GpSuc1b*, *GpSuc2a*, *GpSuc2b*, and *GpSuc2c* were designed (Table 1). Genomic PCR was carried out and the PCR products were purified using a DNA purification kit (Promega, Madison, WI, USA), digested with different restriction enzymes and ligated into a His tag-holding expression vector pET-24b, resulting in the recombinant expression vectors. The resultant plasmids were confirmed by sequencing and transformed into *E. coli* strain BL21 (DE3) cells. The transfected cells were grown at 37 °C in Luria–Bertani medium containing 50 μg/mL kanamycin, and then induced with 0.5 mM isopropyl *β*-D-1-Thiogalactopyranoside (IPTG) for 20 h at 16 °C. Transformation and purification of recombinant BmSUC1 were simultaneously carried out. The soluble proteins in the supernatant were separated from the precipitate after ultrasonic cytolysis and centrifugation. Purification of the recombinant proteins was achieved using a Ni-NTA affinity column (Qiagen, Hilden, Germany). The concentrations of proteins were measured using the BCA Proteins Assay Kit (Sangon Biotech, Shanghai, China). Enzymatic activity of purified proteins was determined using sucrose as substrate according to the same method as described above. To establish the effect of pH on the sucrose hydrolytic activity, a 200 µL reaction containing 80 μg of total midgut proteins, 100 mM sucrose, and 10 mM Britton–Robinson wide range buffer (pH 4.0–11.0) was incubated for 30 min at 30 °C. Approximately 3 mg of purified recombinant GpSUC1a was collected for the preparation of the rabbit polyclonal anti-GpSUC1a antiserum, as described by Zhu et al. (2013) [21] and Gan et al. (2018) [19].

### 2.6. Expression and Purification of Recombinant Proteins through the Bac-to-Bac System

The ORFs of *GpSuc1a* and *BmSuc1* were subcloned from each pET-24b recombinant vectors and inserted into the donor plasmid pFastBac Dual within *NotI* and *HindIII* sites and transformed to *E. coli* DH10Bac (BmNPV) cells. For the method of culturing *E. coli* DH10Bac cells containing recombinant bacmid baculoviruses baculovirus (please refer to Daimon et al. (2005)) [24]. The white colonies were selected for further amplification. The positive recombinant bacmid baculoviruses confirmed by PCR were isolated from the DH10Bac (BmNPV) cells and transfected into BmN cells using Lipofectamine 2000 (Thermo, Waltham, MA, USA) [14]. The recombinant P0 viral solution was collected from BmN cells 72 h post-infection by centrifuging at 1000× *g* for 5 min. The P0 viral stock was further used to infect BmN cells to generate the P1 virus. The high-titre P2 stock obtained by repeating the steps was used to express recombinant proteins. After 72 h, the nutrient solution was collected and separated into supernatant and sediment by centrifuging at 4000× *g* at 4 °C for 30 min. The recombinant GpSUC1a and the BmSUC1 proteins containing the 6× His-tagged were purified with a Ni-NTA column. The enzymatic activity of purified proteins was determined using sucrose as a substrate with the same method as described above. The primers used are shown in Table 1.

### 2.7. SDS-PAGE and Western Blotting Analysis

To identify the expressional profile of GpSUC1a in larval tissues, total proteins were extracted from midgut, silk gland, fat body, and epidermis of the final instar larvae of *G. pyloalis* as described as above. One microgram proteins were separated by SDS-PAGE (10% (*w*/*v*) polyacrylamide gel) and electroporated onto polyvinylidene fluoride (PVDF) membrane (Millipore, Billerica, MA, USA) with 15 V for 15 min using Trans-Blot (Bio-Rad, Hercules, CA, USA). The membranes were blocked in 5% skim milk for 1 h at room temperature (RT) and incubated with anti-GpSUC1a antibody 1:500 diluted in 5% (*m*/*v*) skim milk in PBST (PBS containing 0.1% Tween 20) overnight at 4 °C. The membrane was washed and incubated with goat anti-rabbit IgG as a secondary antibody 1:5000 diluted in the same blocking buffer for another 1 h at RT then visualized by using an Enhanced HRP-DAB Chromogenic Substrate Kit (Tiangen, Beijing, China). Additionally, expression, purification, and identification of recombinant proteins were analyzed by SDS-PAGE and western blot. In these cases, the anti-His tag antibody was used as the primary antibody in the immunoblot analysis.

### 2.8. Immunohistochemistry Assay

Immunohistochemistry was performed as described by Daimon et al. (2005) [24]. Paraffin sections of *G. pyloalis* midgut (GpMG) were made. The midguts were fixed in Bouin’s fluid (saturated picric acid, formalin, and acetic acid at a ratio of 15:5:1 by volume) at 4 °C for 24 h. Standard histochemical methods were used for tissue dehydration, embedding in paraplast, sectioning into 9-μm thick sections, deparaffinization, and rehydration. The methods for washing and blocking of the midgut slices refer to Daimon et al. (2008) [14]. Additionally, the midgut sections were incubated overnight at 4 °C with the primary antibodies, anti-GpSUC1a serum (1:250). After rinsing 3 times for 10 min each at RT with PBS-Tr, the sections were incubated for 1.5 h at RT with 7.5 μg/mL of the secondary antibodies, Alexa Fluor 488-labeled goat anti-rabbit IgG F (ab)2 fragment (1:200, Sangon Biotech, Shanghai, China). The slides were counterstained with a 4′,6-diamidino-2-phenylindole dihydrochloride solution (DAPI). The fluorescence was observed under a fluorescence microscope (Olympus BX53, Tokyo, Japan) and photographed.

### 2.9. Purification of GpSUC1a and BmSUC1 by Immunoprecipitation (IP) and β-FFase Activity Confirmation

To confirm the *β*-FFase activity of GpSUC1a and BmSUC1 in vivo, the two proteins were purified from larval midgut using corresponding antibodies and Protein A Agarose (Beyotime, Shanghai, China). In brief, a total of 200–1000 μg midgut proteins were transferred to a fresh, pre-cooled microcentrifuge tube containing 20 µL of Protein A Agarose bead slurry and rocked gently for 1 h at 4 °C to remove non-specific binding. The supernatant collected (0.22 μg) by pulsing in a centrifuge (5 min, 2500× *g*, 4 °C) was added to corresponding antibodies or rabbit IgG and rocked gently overnight at 4 °C. The immune complex was captured by adding 40 µL of Protein A Agarose bead slurry and shaking for 3 h at 4 °C. The agarose beads were obtained by centrifuging at 2500× *g* for 5 min at 4 °C and washing six times with the pre-cooled wash buffer in One Step Animal Tissue Active Protein Extraction Kit. Additionally, purification of GpSUC1a and BmSUC1 were analyzed by SDS-PAGE and western blot. The enzymatic activity of purified proteins was determined using substrate of sucrose or maltose. The method was the same as described above in the case of using sucrose as a substrate. However, the maltose substrate was incubated with a 0.4 M PBS buffer (pH 6.0) for 10 min at 30 °C. The content of glucose generated by the reaction was detected with a Glucose Detection Kit (Jian Cheng, Nanjing, China), and the activity of the enzyme was calculated.

### 2.10. Liquid Chromatograph-Mass Spectrometer (LC-MS) Analysis

To perform an IP and an LC-MS analysis, GpSUC1a and BmSUC1 were purified from larval midgut and verified using SDS-PAGE as described above, and the fractions containing purified protein were recovered. The purified proteins were then digested using a 40 μL Trypsin buffer (Sigma, catalog number: T4049) at 37 °C for 16–18 h. Finally, peptides were extracted, concentrated to dryness under a vacuum, and stored at −20 °C until LC-MS analysis. Post-translational modifications of protein such as N-glycosylation, phosphorylation, methylation, acetylation, and ubiquitination were identified by LC-MS analysis using an Easy nLC 1000 coupled to a Thermo Scientific Orbitrap Fusion mass spectrometer (Thermo Fisher Scientific, San Jose, CA, USA). Each fraction was reconstituted in 0.1% formic acid and analyzed in liquid chromatography. Samples were loaded on to a 75 μm diameter chromatographic column (length 150 mm) packed with 3 μm Reprosil Pur C18 AQ resin. Solvents A and B were 0.1% formic acid aqueous solution and 0.1% formic acid acetonitrile solution, respectively. The gradient was 5% to 10% B in 16 min, 10% to 22% B in 35 min, 22% to 30% B in 20 min followed by 30% to 95% B in 1 min, and 95% B for 6 min. The flow rate was set to 600 nL/min. Mass spectra were extracted, deconvolved using Proteome Discoverer 1.4.1.14, and searched against a concatenated database using Mascot 1.4. peptides by allowing a maximum of two trypsin missed cleavages with a mass tolerance of ±15 ppm and a fragment ion mass tolerance of ±0.02 Da. Only peptide scores higher than 10 were retained for analysis.

## 3. Results

### 3.1. GpSuc1a Is a Homologous Gene of BmSuc1 Identified in G. pyloalis

**Figure 1:**
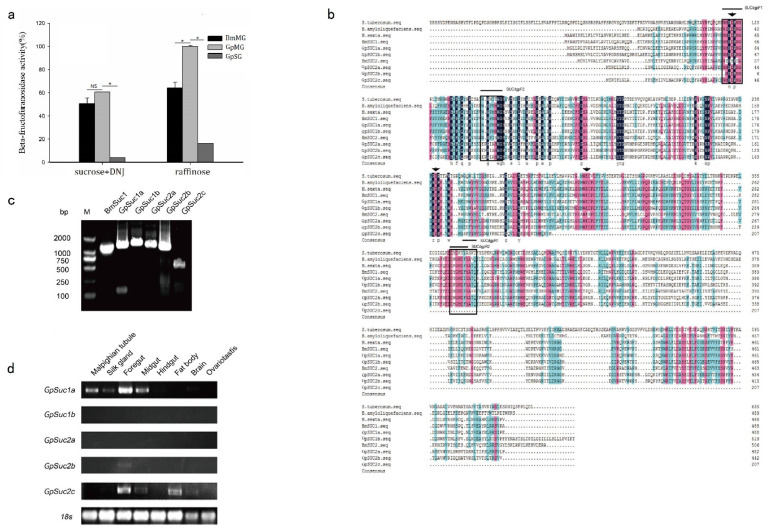
*BmSuc1* homologous gene *GpSuc1* was identified in *G. pyloalis*. (**a**) *β*-FFase activity in different tissues (BmMG, *B. mori* midgut). *β*-FFase activity in BmMG and GpMG were significantly upregulated compared with control. Bars represent the mean ± SEM (*n* = 3) of at least three independent experiments performed in triplicate. The asterisk represents a significant difference (two-way ANOVA, followed by Tukey’s test as post hoc, 0.01 *≤* * *p ≤* 0.05) (the enzymatic activity of β-FFase in the midgut of *G. pyloalis* was taken as 100% when the raffinose was used as substrate). (**b**) Multiple sequence alignment of the *β*-FFase protein from *G. pyloalis* with other *β*-FFase proteins. Amino acids conserved among 100%, ≥75%, and ≥50% were highlighted in black, pink, and blue, respectively. The arrows represented conservative enzyme active sites, and the boxes indicate degenerate primers sites. GenBank accession numbers are as follows: *S. tuberosum*, ACC93584; *B. amyloliquefaciens*, YP008422504; *M. sexta*, ACX49763; *B. mori*, AB366559. Amplification and tissue expression patterns of *β*-FFases in the *G. pyloalis* larval tissues. (**c**) Genomic PCR amplified open reading frame of *BmSuc1*, *GpSuc1a*, *GpSuc1b*, *GpSuc2a*, *GpSu2b*, and *GpSuc2c*. (**d**) Expression pattern of *β*-FFase in *G. pyloalis* larvae. The 18s rRNA gene was used as control.

The enzymatic activities of *β*-FFase in vivo were detected using sucrose adding DNJ or raffinose as substrates (Figure 1a). As shown in Figure 1a, GpMG could decompose sucrose, and it wasn’t affected by DNJ. Although raffinose could not be digested by α-glucosidase, it could be hydrolyzed by GpMG. However, neither sucrose nor raffinose could be hydrolyzed by the *G. pyloalis* silk gland (GpSG).

The specific primers were designed based on the β-FFase sequence of *Solanum tuberosum* (*S. tuberosum*, ACC93584), *Bacillus amyloliquefaciens* (*B. amyloliquefaciens,* YP008422504), *Manduca sexta* (*M.* *sexta,* ACX49763) and *B. mori* (AB366559) (Figure 1b). Degenerate PCR and RACE experiments were used to clone the *BmSuc1* homologous genes in *G. pyloalis*, including *GpSuc1a* (MN365898), *GpSuc1b* (MN380475), *GpSuc2a* (MN380476), *GpSuc2b* (MN380477), and *GpSuc2c* (MN380478). The characteristics of the *β*-FFase amino acid sequence of *G. pyloalis* are shown in Table 2. According to their ORF sequences, we amplified *GpSuc1a*, *GpSuc1b*, *GpSuc2a*, *GpSuc2b*, *GpSuc2c*, and *BmSuc1* using genomic DNA (Figure 1c). To further analyze the expression pattern of *β*-FFase in the different tissues of *G. pyloalis*, a semi-quantitative RT-PCR was performed. As shown in Figure 1d, *GpSuc1a* and *GpSuc2c* highly expressed in various tissues, including malpighian tubule, silk gland, foregut, midgut and fat body. However, the expression of *GpSuc1b*, *GpSuc2a*, and *GpSuc2b* was not detected in these tissues.

Amino acid sequence alignment analysis showed that these five genes were homology of the *β*-FFase in *S. tuberosum* (ACC93584), *B. amyloliquefaciens* (YP008422504), *M. sexta* (ACX49763), and *B. mori* (AB366559), which belong to 32 glycosyl hydrolase family (Figure 1b). The GpSUC1a, GpSUC1b, GpSUC2a, and GpSUC2b sequences contain three conservative domains similar to BmSUC1. In addition, GpSUC2c contains an pretermination stop codon.

### 3.2. Recombinant GpSUC1a Showed Lower Activity Compared to BmSUC1

**Figure 2:**
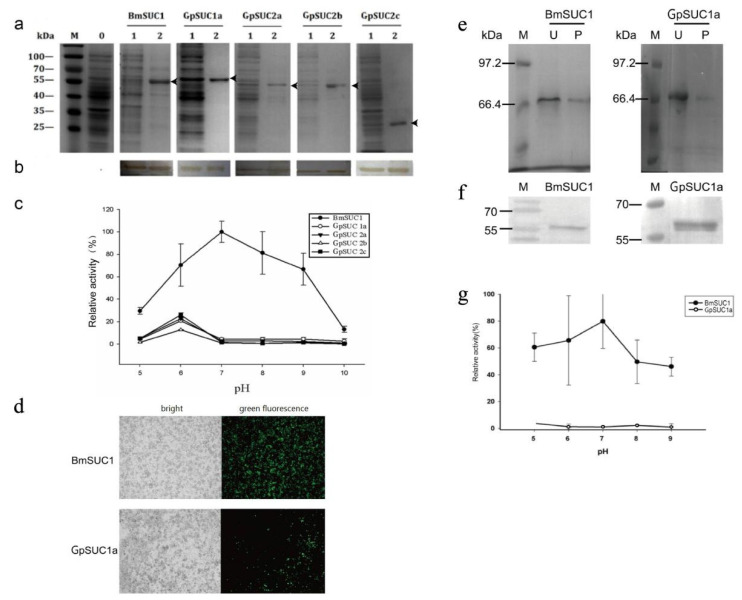
Expression of recombinant proteins in vitro. SDS-PAGE (**a**) and western blot (**b**) were used to analyze the recombinant proteins. Lanes 0: empty pET-24b vector, Lanes 1 total protein induced by 0.5 mM IPTG, Lanes 2: recombinant protein. Arrowheads indicate the recombinant protein. (**c**) Enzyme activity assays of recombinant proteins. Data are mean ± SEM (*n* = 3) (The enzymatic activity of BmSUCl at pH 7.0 was taken as 100%). (**d**–**f**) The recombinant proteins BmSUC1 and GpSUC1a were expressed in the Bac-to-Bac/BmNPV/BmN cell. (**d**) The BmN cells were infected by P2 virus under the fluorescence microscope (10×). The recombinant proteins BmSUC1 and GpSUC1a were analyzed by SDS-PAGE. (**e**) (U, unpurified products; P, purified proteins) and western blot (**f**). (**g**) Enzyme activity assays of the recombinant proteins BmSUC1 and GpSUC1a, which were expressed in the Bac-to-Bac system. Data are mean ± SEM (*n* = 3) (The enzymatic activity of BmSUCl at pH 7.0 was taken as 100%).

The recombinant proteins were expressed and purified from prokaryotic expression system. The specific primers for cloning *GpSuc1a*, *GpSuc1b*, *GpSuc2a*, *GpSuc2b, GpSuc2c* and *BmSuc1* ORFs are shown in Table 1. And these target sequences were cloned into pET-24b expression vectors. The positive recombinant vectors, confirmed by double digestions (Supplementary data, Figure S1), were transformed into *E. coli* strain BL21 (DE3) cells to express recombinant proteins. The result indicated that all recombinant proteins except GpSUC1b could be expressed in DE3 cells (Supplementary data, Figure S2). The SDS-PAGE results showed that the positive recombinants were obtained and identified using western blot (Figure 2a,b). Purified proteins were used for enzymatic property analysis. The enzymatic activities of the recombinant proteins at different pH (5.0–10) in vitro were analyzed (Figure 2c). The results showed that pH 7.0 was the optimal condition for BmSUC1, but the recombinant GpSUC1 and GpSUC2 had little enzymatic activities.

Recombinant proteins GpSUC1a and BmSUC1 were expressed in the Bac-to-Bac/BmNPV/Bm cell system. After adding the P2 virus for 72 h, the fluorescence signal in Figure 2d showed that the recombinant baculovirus was successfully infected and the recombinant protein was successfully expressed as analyzed by the SDS-PAGE (Figure 2e) and the western blot (Figure 2f). The enzymatic characterization of purified protein BmSUC1 and GpSUC1a were determined at different pH conditions (5.0–9.0.) (Figure 2g). Similarly, GpSUC1a also showed little activity compared to BmSUC1.

### 3.3. GpSuc1a Highly Expressed in the Midgut of G. pyloalis and Displayed obvious β-FFase Activity

**Figure 3:**
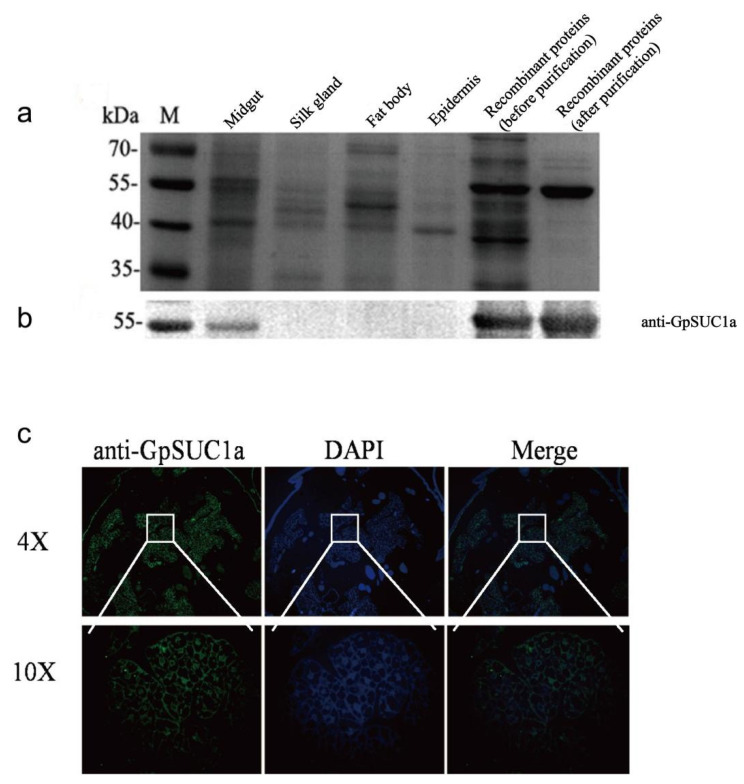
Expression pattern and localization of *GpSuc1a* protein. Expressional patterns of GpSUC1a in different tissues by SDS-PAGE (**a**) and western blot (**b**) analysis. (**c**) Immunohistochemistry analysis of *GpSuc1a* protein in the midgut. The midgut sections incubated with the secondary antibody labeled with IgG/Alexa Fluor 488 (green) and DAPI (blue) as overlapping signals.

To identify GpSUC1a protein in larval tissues, an SDS-PAGE, a western blot, and an immunohistochemistry assay were performed (Figure 3) by using an anti-GpSUC1a antibody. The results showed that GpSUC1a was expressed only in the midgut (Figure 3a,b). The immunohistochemistry assay further characterized the localization of GpSUC1a in the midgut of *G. pyloalis* (Figure 3c).

**Figure 4:**
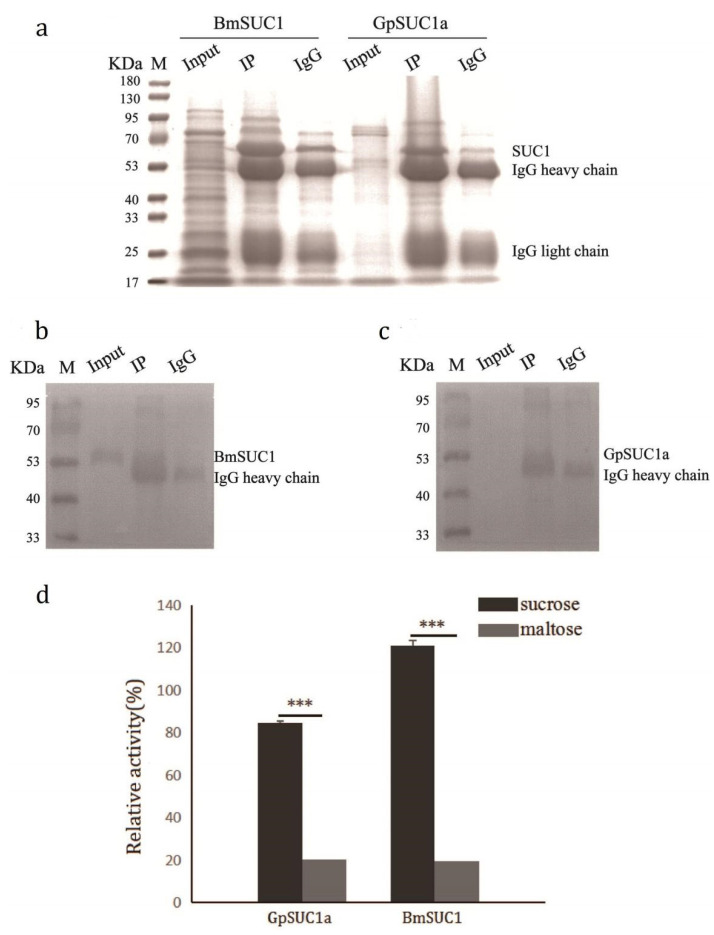
Confirmation of *β*-FFase activity in the larvae of GpSUC1a purified from the midgut. SDS-PAGE (**a**) and western blot (**b**,**c**) were used for analyzing the GpSUC1a in vivo. (**d**) Enzyme activity assays of the proteins in vivo. (Input: One Step Animal Tissue Active Protein Extraction Kit; IP: Purified protein with corresponding antibody added; IgG: Normal Rabbit IgG). Data are mean ± SEM (*n* = 3, *** *p* < 0.001).

Next, we purified GpSUC1a and BmSUC1 proteins from *G. pyloalis* and *B. mori* larval midguts, respectively, by IP experiment (Figure 4) and then the purified proteins were analyzed by SDS-PAGE and western blot (Figure 4a,b). The results of enzymatic characterization showed that GpSUC1a displayed a high sucrose hydrolase activity similar to that of BmSUC1 (Figure 4c); but, none of them could catalyze the hydrolysis of maltose, indicating that both GpSUC1a and BmSUC1 have *β*-FFase activity in vivo.

### 3.4. Identification of Post-Translational Modifications (PTMs) of GpSUC1a and BmSUC1

To compare the PTMs of GpSUC1a and of BmSUC1, two proteins were obtained from larval midguts using an IP assay. After digestion by trypsin and PNGase F, the recombinant proteins were analyzed by LC-MS to detect the post-translational modifications (PTMs) of BmSUC1 and of GpSUC1a.

**Figure 5:**
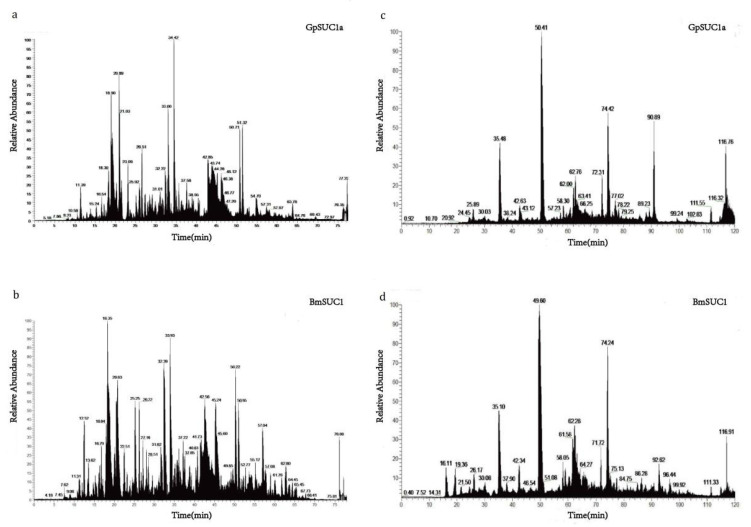
More posttranslational modifications in BmSUC1 than in GpSUC1a. The samples of BmSUC1 and GpSUC1a degradation products were separated by the Easy nLC 1000 system. (**a**) Liquid Mass spectra of GpSUC1a about N-glycosylation and methylation. (**b**) Mass spectra of BmSUC1 about N-glycosylation and methylation. LC-MS results of GpSuc1a and BmSUC1 about phosphorylation, acetylation, and ubiquitination (**c**,**d**).

The GpSUC1a and BmSUC1 degradation product samples were separated by the Easy nLC 1000 system (Figure 5a,b). Both peaks began at approximately 8 min and the whole process stopped at approximately 76 min. After separation, the samples were analyzed by MS using Orbitrap Fusion. N-glycosylation and methylation were identified using Mascot 1.4 combining the raw data with the uniprot-*B. mori* database. The LC-MS results showed that some putative N-glycosylated sites were found in GpSUC1a but none in BmSUC1 (Table 3), and there was more methylation in BmSUC1 than in GpSUC1a. Thus, we evaluated the phosphorylation, acetylation, and ubiquitination using LC-MS (Figure 5c,d). The peaks appeared at 24 and 16 min for GpSUC1a and BmSUC1, respectively, and both disappeared at approximately 118 min. Based on the LC-MS results, these three modifications were found neither in GpSUC1a nor in BmSUC1.

## 4. Discussion

In adaptation of *B. mori* to mulberry, there have been key enzymatic responses to the defensive components in mulberry latex. Mulberry latex contains high concentration of sugar-mimic alkaloids, such as DNJ and D-AB1. These compounds are not toxic to *B. mori*, a mulberry specialist, but they are highly toxic to the larvae of *Samia cynthia* *ricini*, a generalist herbivore [9,25]. This suggests that *B. mori* larvae have adaptive mechanisms that are used against the latex-borne sugar-mimic alkaloids and that allow *B. mori* larvae to absorb the nutrients in mulberry leaves normally. The *BmSuc1* gene is the first animal *β*-FFase-encoding gene to be identified. It is highly insensitive to DNJ and plays a critical enzymatic role in *B. mori* ability to avoid the toxic effects of sugar-mimic alkaloids in mulberry latex [14]. Our previous results indicate that BmSUC1 acts as an essential digestive enzyme in the carbohydrate metabolism of silkworm larvae [19]. Interestingly, *G. pyloalis* is another moth that also feeds on mulberry leaves, and it is not affected by latex and other toxic compounds in mulberry leaves [26]. To explore the five *BmSuc1* homologous genes in *G. pyloalis*. We obtained full-length cDNAs using RACE, then named them: *GpSuc1a*, *GpSuc1b*, *GpSuc2a*, *GpSuc2b*, and *GpSuc2c*, respectively. Bioinformatic analysis indicated that *GpSuc2c* is a gene with an early stop codon, which lacks the central site of the enzyme activity region (-EC-) (Figure 1b).

Our data showed that the *β*-FFase of *G. pyloalis* is similar to the *β*-FFase of *B. mori* in the midgut, which can decompose sucrose in the presence of DNJ but cannot decompose the raffinose (Figure 1a). A tissue expression profile analysis of the five *β*-FFase homologous genes of *G. pyloalis* showed that only *GpSuc1a* and *GpSuc2c* were highly expressed in the midgut, while the other three genes (*GpSuc1b*, *GpSuc2a*, and *GpSuc2b*) were almost not expressed in the midgut (Figure 1d). Our results suggested that *GpSuc1a* is the primary functional *β*-FFase gene in the midgut of *G. pyloalis* because the *GpSuc2c* is a gene with an early stop codon.

Double enzymatic digestion and sequencing showed that the pET-24b *E. coli* recombinant expression vector was successfully constructed (Figure S1). The positive recombinant vector was transformed into *E. coli* BL21 (DE3) cells and induced. The SDS- PAGE and the western blot demonstrated that all recombinant proteins except GpSUC1a were expressed (Figure S2). We could not purify GpSUC1a from the cells transfected with the pET-28a expression vector, most likely due to the presence of more rare codons (CGA, CGG, AGG, AGA, GGA, GGG, AUA, CCC, and ACG) in its sequence.

Although the BmSUC1 and the GpSUC1a could not be expressed as more soluble proteins at 16 °C, the recombinant proteins were expressed mostly in the form of the inclusion body. Failure of the prokaryotic systems to synthesize and correctly fold a significant amount of the full-length fusion protein may be the major reason for the lacking of enzymatic activities in these recombinant proteins. Many eukaryotic proteins that fold correctly in eukaryotes can misfold when expressed in *E. coli* [27,28]. Moreover, in this study, we found that GpSUC1a was highly expressed in vitro, and it was more easily induced by IPTG than others. Thus, *GpSuc1a* may encode the major *β*-FFase gene in *G. pyloalis*. The recombinant proteins were purified, and their *β*-FFase activities were compared. Surprisingly, the recombinant proteins GpSUC1a had lower enzymatic activity compared with BmSUC1 at different pH levels during the enzyme assays (Figure 2c).

Considering the limitations of expressing proteins in prokaryotic systems, the BmNPV/Bac-to-Bac expression system applicable to *B. mori* was used to express the recombinant protein BmSUC1 or GpSUC1a. Compared to the *E. coli* expression system, the BmNPV/Bac-to-Bac expression system has several advantages, such as the capacity for insertion of large DNA fragments, high yields of recombinant protein, and integrated PTMs. It can maintain the structure and the function of natural protein by finishing modifications such as phosphorylation and glycosylation [29,30,31]. However, the GpSUC1a still showed almost no activity when compared with the BmSUC1 enzymatic activities under different pH conditions (Figure 2g).

In order to identify the expression and enzymatic characterization of GpSUC1a in vivo, we confirmed the existence of GpSUC1a in the midgut of *G. pyloalis* by western blot (Figure 3b) and immunohistochemistry (Figure 3c) using the antibodies against GpSUC1a. Importantly, when GpSUC1a was purified from *G. pyloalis* larval midgut, it showed great *β*-FFase activity similar to BmSUC1 (Figure 4). These results indicated that the enzymatic activity of GpSUC1a was reasonably highly detected in the protein purified from the gut, compared with the recombinant protein, suggesting that post translational modification may play an important role. However, the enzymatic activity of GpSUC1a was still lower than that of BmSUC1. The main reason may be the different PTMs for *β*-FFase in *G. pyloalis* and *B. mori*. It is well known that the simplicity of genetic modifications is one disadvantage of the *E. coli* expression system in which PTMs processes could not be finished because of a lack of relevant organelles [32,33,34,35]. As compared to BmSUC1, the expression of GpSUC1a may require more complex modifications. Although the Bac-to-Bac/BmNPV/BmN cell system is superior to the *E. coli* expression system for expression regulation, it is not consistent with the internal environment of the organism. Therefore, it is necessary to use in vivo experiments to distinguish the differences of PTMs between GpSUC1a and BmSUC1.

Known as a pro-protein or a pro-peptide, a protein precursor is an inactive protein (or peptide) that can be turned into an active form by PTMs, such as by breaking off a piece of the molecule or adding another molecule [36,37,38,39]. The identification of PTMs including N-glycosylation, phosphorylation, methylation, acetylation, and ubiquitination in BmSUC1 or in GpSUC1a protein was analyzed to support our hypothesis that GpSUC1a may require more modification. As shown in Table 3, the occurrence of deamidated sites was caused by the digestion of trypsin during mass spectrometry. They were also partly due to the PNGase F enzyme, which could cleave between the innermost GlcNAc and asparagine residues of high mannose, hybrid, and complex oligosaccharides from N-linked glycoproteins. According to the consensus sequence Asn-X-Ser/Thr (N-X-S/T) of N-glycosylation motifs, we found putative N-glycosylated peptides in GpSUC1a but not in BmSUC1 (Table 3), indicating that N-glycosylation of GpSUC1a is more strictly controlled than that of BmSUC1. N-glycosylation is the attachment of the sugar molecule oligosaccharide known as glycan to a nitrogen atom (amide nitrogen of asparagine residue of a protein), which is important for both the structure and the function of some eukaryotic proteins [40,41,42]. Nascimento et al. (2017) found that glycosylated and nonglycosylated Aam1 (the *α*-glucosidase (EC 3.2.1.20) expressed in *Aedes aegypti* larvae midgut) displayed distinct patterns that could influence their catalytic activity [43]. Gonaus et al. (2017) suggested that only the double mutant lacking the N-glycosylation around the active site access (N75 and N175) of AmPDH1 (an oxidoreductase capable of oxidizing a broad variety of sugars) achieved substantially higher electric current than the wild-type enzyme [43]. These data suggest that the activities of GpSUC1a were lower than BmSUC1, whether in *E. coli* or Bac-to-Bac expression systems, due to N-glycosylation. As for methylation, histone methylation has been studied in-depth. However, the regulation processes of other proteins on the occurrence of methylation modification remain unclear. The effect of methylation on the activity of BmSUC1 and GpSUC1a require further study.

Daimon et al. reported that *BmSuc1* showed significant homology with bacterial *β*-FFase, and it belongs to the bacterial lineage [14]. The interaction between intestinal microorganisms and the host in terms of immunity, metabolism, and development is vital to insects. Intestinal microbes can affect the expression of host genes. Sonnenberg et al. (2006) found that colonization of germ-free mice with one of two Bacteroides induced the expression of tumor necrosis factor-*α* (TNF-*α*), which is produced by specific cells of the immune system inflammatory cytokines [39,40]. Regstad et al. (2015) demonstrated that the relative expression of tryptophan hydroxylase 1 (*Tph1*) mRNA in sterile mice was significantly reduced compared with humanized (HM; ex-GF colonized human gut microbiota) mice [43]. Both *BmSuc1* and *GpSuc1a* are derived from bacteria, and the difference in their in vitro activities may be related to intestinal microbes. If the intestinal colony structure of *B. mori* and *G. pyloalis* is different, the expression and the activity of *BmSuc1* and *GpSuc1a* may also be different in their dependence on intestinal microbes. Compared with *BmSuc1*, *GpSuc1a* may need more help; but, it is not clear why the in vitro activity of *BmSuc1* is much greater than that of *GpSuc1a*. This topic needs further research.

In summary, we cloned five *BmSuc1* homologous genes from the final instar larvae of the mulberry pest, the *G. pyloalis*, and analyzed their tissue expression patterns. We identified the *β*-FFase gene in the midgut of *G. pyloalis* (*GpSuc1a*) and confirmed its expression and its location. As *B. mori* and *G. pyloalis* are both insects that feed on mulberry leaves, their *β*-FFase genes should have the same biological function in the body’s defense system against mulberry sugar-like alkaloids. However, our comparative studies have shown that *GpSuc1a* and *BmSuc1* have significant differences in terms of expression conditions in vitro and PTM in vivo, showing that the homologous genes have the characteristics of biological diversity.

## 5. Conclusions

In this study, we completed the report on the characterization of *β*-FFase genes from *G. pyloalis* for the first time. Moreover, this is the first comparison of expression regulation between two mulberry feeding insects, *B. mori* and *G. pyloalis*. The *β*-FFase activity in the midgut of *G. pyloalis* larvae and purified GpSUC1a from the midgut were both confirmed, while recombinant GpSUC1a displayed little activity as compared with the higher activity of recombinant BmSUC1. The expression of GpSUC1a was controlled by a post-translational regulation system different from BmSUC1, in which N-glycosylation is necessary and a possible way for *G. pyloalis* to express the functional *β*-FFase gene in vivo. The management of mulberry borers can be inspired by this research.

## Figures and Tables

**Table 1:** The primer sequences used in this study.

Assay	Primer Name	Primer Sequence (5′ to 3′)
Degenerate PCR	SUCdgpF1	TGGATIAAYGAYCCIAAYGG
	SUCdgpR1	GTGIGTIGCRTARAAITC
	SUCdgpF2	TGGGGICCIATGCAYTGGGGICA
	SUCdgpR2	GCRTARAARTCRTGICCRTRRTC
RACE	*GpSuc1a*-5RAgsp	AGCACCACCGGAGAAACACATTTCTT
	*GpSuc1a*-5RAngsp	GGGCTGGACACGTGGCCCCAGTGCAT
	*GpSuc1a*-3RAgsp	ACAGTTTACATGTGGGAATGCCCTGA
	*GpSuc1a*-3RAngsp	GGCATGGAACCCAAAGGCGATCGGTT
	*GpSuc1b*-5RAgsp	TCCACTGCCCGAGAAGCACTGTTCTT
	*GpSuc1b*-5RAngsp	GCGCTGGACGAGTGCCCCCAATGCAT
	*GpSuc1b*-3RAgsp	ATGGGGTACATGTGGGAGTGCCCTGA
	*GpSuc1b*-3RAngsp	GGAATGGCACCTCAGGGTGACAGGTA
	*GPSuc2a-*5RAgsp	AGCAATTGGTAGATGCTTCCAATGGAA
	*GpSuc2a*-5RAngsp	ATGCCCCCAATGAGCTATTCCAGGTT
	*GpSuc2a-*3RAgsp	GATGGCGTCACTACTAAAAAGTATCGG
	*GpSuc2a-*3RAngsp	CGCCTCAGGGTATAGAACCGGAAGGA
	*GpSuc2b-*5RAgsp	CTCCACTTTTGTCATATGCCTTGTCC
	*GpSuc2b-*5RAngsp	TCGCTGCTCATGGTCGGGCTTTTTG
	*GpSuc2b-*3RAgsp	CTTGGATACATGTGGGAATGTCCAGATT
	*GpSuc2b-*3RAngsp	CGCCTCAAGGTGTGAAACCTGAAGGA
	*GpSuc2c-*5RAgsp	TCTGTGACTTATGTGCCAAAATGCTG
	*GpSuc2c-*5RAngsp	TTAAGGGAATGCCATTCTCATCCGTT
	*GpSuc2c-*3RAgsp	CTTGGMTAYATGTGGGAATGTCCAGATT
	*GpSuc2c-*3RAngsp	CAAATGACAAAACAAACTGGCAAGAA
RT-PCR	*GpSuc1a* Fw	ATTGTTGTACACTGGGCGACTAACC
	*GpSuc1a* Re	TGGTGAGTCGTTCGGAGTGTACGTA
	*GpSuc1b* Fw	CGCAGCTATGAACAACTTGAGGC
	*GpSuc1b* Re	CTGACCACCACCATTTGTGCCAGT
	*GpSuc2a* Fw	GATGGTACAGGTGGGGATGATG
	*GpSuc2a* Re	CAGTGCTCCTTATTCCATCATGG
	*GpSuc2b* Fw	TGTCAGGGAACTTCGGTTGAAC
	*GpSuc2b* Re	GCCATTGAAAATACTTTTGGCGACT
	*GpSuc2c* Fw	CGAAGAGCACCAAGCTCTTGGTGTG
	*GpSuc2c* Re	TTGATTCATATAGTAGAACCTGAC
	*BmSuc1* Fw	CGGACCCGTTTTACAACGAA
	*BmSuc1* Re	CACGTAGGAGAGGACTGGAT
Genomic PCR And pET-24b Expression Vectors Construction	*GpSuc1a* F	ATA**GGATCC**GATGGGGCTCCTAAGGCTAATCG
	*GpSuc1a* R	CCG**CTCGAG**TTCAGGTATACTTCTTCTTAAATG
	*GpSuc1b* F	CTAGATCT**CATATG**ATGGCGGTCTTCACATTCACTG
	*GpSuc1b* R	CCG**CTCGAG**CAATGTTGGGATAACTGG
	*GpSuc2a* F	GGACTTC**CATATG**ATGACGAGAGATTTACTGAGGC
	*GpSuc2a* R	TAT**CTCGAG**GGGCAAAACGGAACGCTTGAG
	*GpSuc2b* F	CGC**GGATCC**GATGAATTGATCCGAACGGCTTCAG
	*GpSuc2b* R	TATCAAAT**GCGGCCGC**AACAACAGAACGTGTCAATG
	*GpSuc2c* F	ATA**GGATCC**GATGACTAAAACTTTTTTAGGGC
	*GpSuc2c* R	CCG**CTCGAG**ATTTACTTCTTGCCAGTTTG
	*BmSuc1* F	CAGCTGTA**CATATG**TTCGCCTGGAGCACAC
	*BmSuc1* R	CGG**CTCGAG**AGCGGGTACACTTCTTCTCAATC
pFastBac Dual Expression Vectors Construction	GFP F	CCG**CTCGAG**ATGGTGAGCAAGGGC
	GFP R	CGG**GGTACC**TTACTTGTACAGCTC
	pFB-BmSuc1 F	ATTT**GCGGCCGC**TATGTTCGCCTGGAGC
	pFB-BmSuc1 R	CCC**AAGCTT**TTAATGATGATGATGATGATGAGCGGG
		TACACT
	pFB-GpSuc1a F	ATA**GGATCC**GATGGGGCTCCTAAGGCTAATCG
	pFB-GpSuc1a R	CCC**AAGCTT**TTCAATGATGATGATGATGATGGGTATA
		CTTCTTCTTAAATG

Bold nucleotides indicate restriction sites.

**Table 2:** Characteristics of β-FFase amino acid sequences of *G. pyloalis*.

Protein	Mass (Da)	PI	Homology (%)			
			BmSUC1 BmSUC2	*M. sexta*	*B. amyloliquefaciens*	*S. tuberosum*
GpSUC1a	56,400.10	4.48	64.90	65.24	37.86	22.26
			35.98			
GpSUC1b	58,635.53	5.31	54.89	60.27	35.74	21.70
			35.03			
GpSUC2a	55,794.92	5.47	37.43	41.27	37.90	18.68
			35.26			
GpSUC2b	50,642.75	6.40	37.50	38.97	37.20	19.62
			34.50			
GpSUC2c	23,868.99	7.78	16.73	16.57	16.70	10.19
			13.28			

**Table 3:** Comparative identification results of BmSUC1 and GpSUC1a about N-glycosylation and methylation.

Protein	Position (aa)	Amio Acid Sequences	Modifications
BmSUC1	21–47	ALRQQNETTKRELEEYIADKKA	Methyl [K20]; Deamidated [R11]
		EINPR	
GpSUC1a	20–45	SFKQQFDNVADLEEYIAQKRTE	Deamidated [Q5; Q18;R20;R26]
		INPR	
BmSUC1	48–73	YRPHYHISPPVGWMNDPNGFS	Methyl [R2; K24; K26];
		YYKEK	Deamidated [R2; N18]
GpSUC1a	46–71	YRLQYHVTPPVGWMNDPNGFS	Deamidated [Q4]
		FYKGE	
BmSUC1	162–183	KYEGNPVLSYVPDNSADFRDPK	Methyl [R19; K22]
GpSUC1a	160–181	KYEGNPVLTYTPRPDFNDSDPK	Methyl [R19; K22];
			Deamidated [N13]
BmSUC1	187–202	FKDHWYVVIGSSSNK · R	Methyl [K2]; Deamidated [N14]
GpSUC1a	185–201	HEDHWYVVIGSKTVDGR	No sites
BmSUC1	283–307	TDKYFQELDYGHDFYAT	Methyl [K3; K25]
		QTIQGDGK	
GpSUC1a	282–306	PETEFQELDYGHDIYATQSLEK	No sites
		DGT	
BmSUC1	337–344	ELQLIGTR	No sites
GpSUC1a	336–343	EIKLEGDR	Methyl [K3]
BmSUC1	360–376	SVHNGDLEPQQAIEFGP	No sites
GpSUC1a	359–375	SLFDGDLLPEQSIEFEK	Deamidated [Q11]
BmSUC1	424–436	QVEWVPIGKTSWR	No sites
GpSUC1a	359–375	QVEWNPIGSQSWR	Deamidated [Q1]
BmSUC1	467–483	VKNSSPQTLSVEAYRLR	Deamidated [Q7]
GpSUC1a	467–483	LTNLSPQNLSVEAYHLR	Deamidated [N3; N8]
BmSUC1	484–488	RSVPA	No sites
GpSUC1a	484–505	RSIPEMDVFVTITENRLNSGFK	Deamidated [R1; N18]

The yellow and blue labels correspond to methyl and deamidated sites, respectively, while the green was both. The red labels represented putative N-glycosylated sites.

## Data Availability

The data presented in this study are available on request from the corresponding author.

## Supplementary Materials

The following supporting information can be downloaded at: https://www.mdpi.com/article/10.3390/insects13050410/s1, Figure S1: Recombinant vectors digested by restriction endonucleases; Figure S2: SDS-PAGE and western blot analysis of recombinant proteins at different inducing conditions.
