# Supplementary material for: A Comparative Analysis of Bombyx mori (Lepidoptera: Bombycidae) β-fructofuranosidase Homologs Reveals Different Post-Translational Regulations in Glyphodes pyloalis Walker (Lepidoptera: Pyralidae)
Source: Insects. 2022 Apr 26;13(5):410. doi: 10.3390/insects13050410 (PMC9143633; doi:10.3390/insects13050410)
Supplement: Supplementary file 1 [file insects-13-00410-s001.zip › insects-1640776-supplementary.pdf]

Supplementary data

FigureS1.

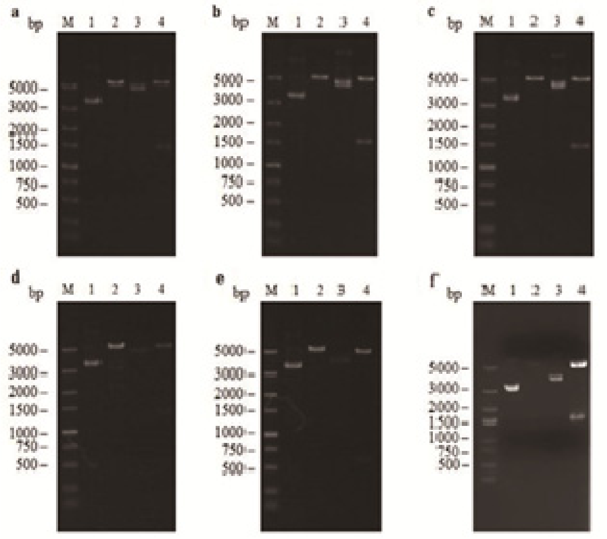

Figure S1 Recombinant vectors digested by restriction endonucleases. M to lane 4 were DNA marker, empty pET-24b vector, the digested pET-24b vector, recombinant vector, and the digested recombinant vector respectively. (a) to (f) were recombinant plasmid pET-24b/*GpSuc1a,* pET-24b/*GpSuc1b*, pET-24b/*GpSuc2a*, pET-24b/*GpSuc2b*, pET-24b/*GpSuc2c*, and pET-24b/*BmSuc1*.

FigureS2.

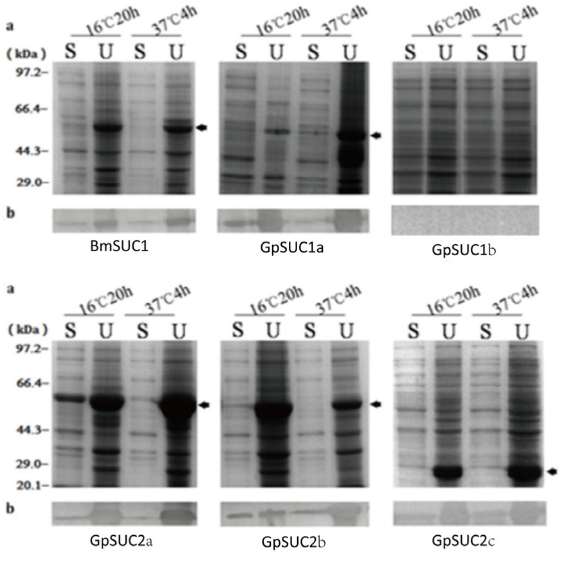

Figure S2 SDS-PAGE (a), (c) (S, supernatant component; U, unsolubilized component) and western blot analysis (b) of recombinant proteins at different inducing conditions.SDS-PAGE and western-blot results showed that the soluble expression of recombinant proteins verified by colony PCR was higher at 16°C for 20 h compared with 37°C for 4 h with 0.5 mM IPTG induction .
